# Healthcare Providers' Knowledge and Current Practice of Pain Assessment and Management: How Much Progress Have We Made?

**DOI:** 10.1155/2016/8432973

**Published:** 2016-11-14

**Authors:** Khawla Nuseir, Manal Kassab, Basima Almomani

**Affiliations:** ^1^Faculty of Pharmacy, Jordan University of Science and Technology, Irbid, Jordan; ^2^Faculty of Nursing, Jordan University of Science and Technology, Irbid, Jordan; ^3^Faculty of Health, University of Technology, Sydney, NSW, Australia; ^4^Faculty of Nursing, University of Western Sydney, Sydney, NSW, Australia

## Abstract

*Context*. Despite improvement in pain management and availability of clinical treatment guidelines, patients in Jordan are still suffering from pain. Negative consequences of undertreated pain are being recognized as a reason for further illnesses and poor quality of life. Healthcare providers (HCPs) are responsible for relieving pain of their patients.* Objective*. To evaluate the knowledge and attitudes of HCPs toward pain management in Jordan.* Methods*. A 16-item questionnaire with agree or disagree options was given to 662 HCPs in seven hospitals in Jordan who volunteered to participate in the study. Following data collection, the responses were coded and entered into SPSS.* Results*. There was a statistically significant difference (*p* < 0.004) in percentage scores between physicians (36%) and pharmacists (36%) versus nurses (24%). The level of knowledge was the best among physicians, followed by pharmacists specifically in the area of cancer pain management. Nurses scored the lowest for knowledge of pain assessment and management among HCPs. However, HCPs overall scores indicated insufficient knowledge specifically in relation to pain assessment and management among children.

## 1. Introduction

Pain is often undertreated even though it is the main reason for people seeking medical advice [1–3]. The result is needless suffering and complications that cause further injury and disability [3–8].

Pain management is an integral part of healthcare professionals' (HCPs') practices, those dealing with pain on daily basis. Thus for effective pain management HCPs must be well-educated and knowledgeable about pain. This begins with a thorough and accurate assessment of patient's pain [2, 9]. Screening for pain should be a part of a routine assessment, and this has led the American Pain Society (APS) to declare pain as the “fifth vital sign” [10]. Worldwide, surveys of HCPs continue to reveal a deficit in their knowledge. Furthermore, findings of research emphasized the need to improve education and to conduct training programs about available pain management options to improve provided care and life quality of patients. There are many possible barriers to effective pain management among HCPs. Misconceptions and myths about pain and pharmacological pain treatment, particularly fear of opioids addiction as well as serious adverse effects like respiratory depression, are blamed for pain undertreatment [11–13]. In addition lack of knowledge about dealing with special groups of patients such as the elderly and the very young led to undertreatment or even no treatment of pain in such cases [4, 14–17].

Numerous studies were done to assess HCPs' knowledge and attitudes about pain. Mostly what they show was that in general many HCPs, especially nurses, lack adequate knowledge about pain, which led to undertreatment of pain [13, 18–23]. Studies had also shown that HCPs bear misconceptions and myths about pain that impede proper pain management [22, 24–27].

Pain management knowledge deficiency is not the only factor that affects patients' well-being. Nurses' perceptions and satisfaction regarding pain management toward postsurgery patients were low in a study conducted in Iran [28]. Perceptions about spirituality and spiritual care of the patients is another factor to be taken in consideration [29]. Healthcare system was also cited as one barrier for better pain management for the elderly in multicenter study in Poland [17].

Pain is a global health issue that requires the attention of the health community [30]. Pain management is complex and multifactorial; thus a deeper understanding of the barriers for proper and optimum pain management needs to be addressed in order to remedy the deficiencies among HCPs and improve patient care. Education and training of HCPs is an important intervention that improves their skills which reflects positively on the patients [31–34].

Physicians knowledge and attitudes about pain have been explored also, although perhaps to lesser extent than nurses' [13, 20, 23, 26, 35–38]. Even fewer studies dealt with the role of pharmacists, particularly clinical pharmacists in pain management [21, 39–41]. This is probably because the discipline of clinical pharmacy is relatively new. In Jordan clinical pharmacists as well as PharmDs (doctor in pharmacy degree) are just starting to have an effective role in clinics and health centers as well as hospitals. They are the drug experts and their knowledge about pain needs to be assessed further.

In Jordan and to the best of our knowledge, pain management does not comply with international guidelines and adequate assessment tools are rarely found at clinics or hospitals. Recently a study conducted which evaluated nurses' knowledge and attitudes regarding pain treatment in Jordan showed that Jordanian nurses from four hospitals centers have a lower level of knowledge and attitude regarding pain management [42] compared to nurses from other studies; this might be due to deficit in pain education and training during undergraduate studies. Another study done by Kassab et al. showed a deficit in nurses' knowledge about pain suffered by infants as well as lack of understanding of assessment and management for this special group of patients [43]. On the other hand, research that explores HCPs attitudes and knowledge toward pain, pain assessment, and pain management is sparse in our region; in Jordan only nurses knowledge and attitudes were explored [20, 22, 26, 44].

Pain management should be a multidisciplinary effort and the responsibility of all HCPs. As such, this study was designed to explore the knowledge and attitudes of different HCPs. This work intends to report and reflect current knowledge and practice toward pain in a wide sample of hospitals throughout Jordan including doctors, pharmacists, and nurses. Additionally, pharmacists' role in supporting and educating other HCPs regarding pain management is worth investigating, but first their knowledge and attitudes must be studied.

## 2. Methods

### 2.1. Sample

A cross-sectional survey was carried out at several hospitals (*n* = 15) located at different regions of Jordan (northern, central, and southern) from 2012 to 2013. Participants targeted for this study were physicians (consultant and resident), pharmacist (PharmDs and clinical pharmacist), and nurses (registered, diploma, and midwives). Jordan University of Science and Technology Institutional Review Board (IRB) approved this research.

### 2.2. Questionnaire

The questionnaire was comprised of 16 items in English, and participants were asked to reply according to five-point Likert scales ranging from “strongly agree” to “strongly disagree.” Answers were evaluated considering the extent to which they were compatible with pain therapy standards commonly acknowledged by the international pain management guidelines. The choice of the items was influenced by the survey of Zanolin et al. and piloted survey carried out in one hospital in Jordan by a small number of HCPs (*n* = 15; 5 physicians, 5 nurses, and 5 pharmacists). The data from the pilot participants were not included in the final analysis. The pilot study resulted in very minor modifications, such as clarifying the meaning of a word by inserting a synonym next to the original word.

Questionnaire consisted of three sections. The first section collected demographic data. The second section focused on HCPs' knowledge about pain and pain management, and the third section focused on current practices.

### 2.3. Data Collection

A self-completed questionnaire was distributed to the HCPs in the participating sites. The research assistant visited the same hospital several times over days until all available and willing HCPs were approached. Willing Participants were included in the study if they signed consent form and filled the questionnaire. Participants' personal information was kept anonymous; however, they were only asked to include their qualifications and the name of their departments. They were instructed to complete questionnaire at same setting and without consulting medical texts.

### 2.4. Statistical Analysis

Descriptive analyses were used to summarise the data of the total sample. Continuous data was expressed as means ± standard deviation while categorical data was expressed as numbers and percentages. Categorical variables were analysed using the Chi-square (*χ* ^2^) test or Fisher's exact test as appropriate. In order to analyse associations between HCPs' knowledge and the possible influence, binary logistic regression was performed with an odds ratio (OR) and 95% confidence intervals (CI). All tests were two-sided, and a *p* value < 0.05 was deemed to indicate statistical significance. The answers to 14 different questions for each respondent were computed as categorical variables using a cut-off point for cumulative scores. A respondent was categorized as knowledgeable if the sum of the scores was more than 7 (out of 14) and nonknowledgeable if the sum of the scores was ≤7 (out of 14). Collected data were entered to Statistical Package for the Social Sciences (SPSS, version 19, Chicago, IL, US).

## 3. Results

### 3.1. Sample Attributes

A total of 662 questionnaires were completed, mostly by nurses (60%), physicians (27%), and pharmacists (9%) from different hospitals in multiple areas in Jordan. More than half of the respondents were females (54%) and half of them were young adults (25–35 years old). About 40% of HCPs had qualifications of five years or less in their work and 26% of them were from surgical wards. The demographic characteristics are shown in Table 1.

### 3.2. Knowledge of HCPs

The overall percentage of knowledge of all HCPs was 28.7%. The mean number of total corrected answers was 6.52 ± 2.06 (range 1–13). Surprisingly, no HCP answered all questions correctly. An overview of the questions and their answers is presented in Table 2. Question ten* (for effective treatment of cancer pain it is necessary to continuously assess the pain and the efficacy of the therapy) *was the one with the highest percentage of correct answers (87%) (the correct answer is “agree”). The question with lowest rate of correct answers (18%) was question 5* (staff can always pick up cues from children that indicates that they are in pain) *(the correct answer is disagree).

**Figure 1:**
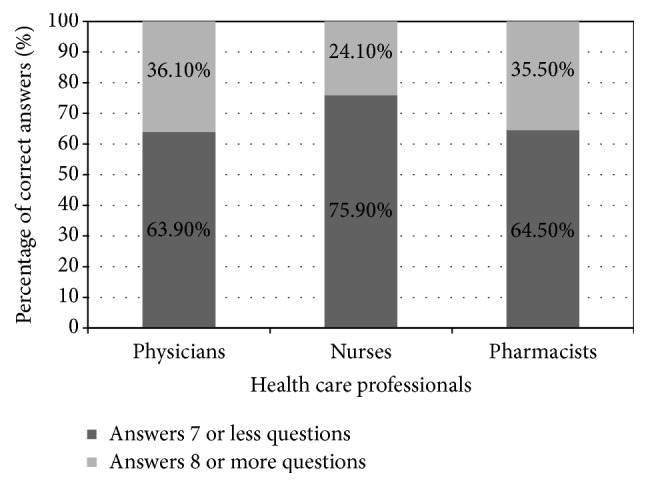


As shown in Figure 1, the data indicated significant difference among healthcare providers in their knowledge (*p* = 0.004). Medical doctors were the most knowledgeable professionals (36.1%, 73/202) followed by pharmacists (35.5%, 22/62) and lastly nurses (24.1%, 95/394). When the mean number of correct answers was calculated, data indicated that doctors had the highest mean (6.90 out of 14) compared to pharmacists (6.56 out of 14) and nurses (6.35 out of 14) Also, there was significant difference (*p* = 0.002) in the level of knowledge between public (26%, 138/531) and private (39.7%, 52/131) hospitals. In addition, level of knowledge was significantly different among different ward types for all HCPs (*p* = 0.034). However, there was no difference in the level of knowledge among different wards within each HCP group.

Two factors (Table 3), having a postgraduate degree and working in private hospital, were significantly associated with higher level of knowledge (OR = 2.005; 95% CI = 1.081–3.720; *p* = 0.027) and (OR = 1.844; 95% CI = 1.099–3.094; *p* = 0.021), respectively. In contrast, being a nurse was significantly associated with lower level of knowledge (OR = 0.315; 95% CI = 0.184–0.537; *p* ≤ 0.001).

### 3.3. HCPs in Clinical Practice

Considering implementation of pain assessment tool, significant difference emerged between the different HCP groups (*p* < 0.001); the highest (92%) were nurses compared to physicians (85%) and pharmacists (65%). Similar trend was observed in regard to application of pain management guideline (*p* < 0.001), nurses 74%, physicians 60%, and pharmacists 47%.

## 4. Discussion

The current study surveyed the knowledge and attitudes of physicians, pharmacists, and nurses toward pain assessment and management in Jordan. Participants' high number as well as the selection of hospitals from different regions of the country rendered the results representative of Jordanian situation. Therefore, findings of this national study could be generalized and give an overview about health care providers knowledge and current pain services at Jordanian hospitals. Furthermore, questionnaires exploring knowledge and attitudes are considered good predictors of good pain management practices [45].

The overall percentage of knowledge among all HCPs was 29% with nurses scoring the lowest. These results are in agreement with outcomes of several other similar studies around the world [19, 20, 26, 36, 46–49]. One of these studies, which inspired this work, found that the overall correct answers of all HCPs was 50%; moreover, they also discovered that nurses scored less than physicians (47.6% versus 57.3%) [19]. Furthermore comparable results emerged from a study that explored knowledge and attitudes of nurses in Jordan, at which the average score was (19.3 out of 40) [42]. The latter surveyed nurses' knowledge and attitudes toward pain management, using a 40-item questionnaire (KAS) and was restricted to certain wards in four hospitals in Jordan. In the current study we aimed at investigating the knowledge and attitudes of all HCPs at multiple sites using a simple 16-item questionnaire. Nevertheless, comparable deficiencies in knowledge and practices of nurses confirmed the conclusions.

The present study found a statistically significant difference between percentages of correct answers among the different professional groups (physicians (36%) and pharmacist (36%) versus nurses (24%)). This variation was also noticed by other conducted studies [21, 23, 35, 41, 50] and could be due to inadequate educational curriculum and insufficient clinical training programs [23, 26, 44, 51].

The main lack of knowledge and inappropriate practice in this study were shown primarily in the following areas: recognizing the difference between tolerance and dependence (q3) and pain and pain management in children (q5 and q6). Also there is deficit in pharmacology of pain medications particularly narcotics (q7 and q8). Differentiation between chronic and acute pain is another area of poor knowledge (q14). These shortages are similar to other studies [19, 23, 35, 36, 39]; this indicates a worldwide weakness of pain management knowledge among HCPs that needs to be addressed globally.

The difference in utilization of pain tools among health care providers in different specialist fields was significant from a statistical point of view, nurses being the chief users followed by doctors then pharmacists. This finding could be explained by the fact that nurses devote more time to their patients compared to other care providers. This was in agreement with other studies that showed nurses were better at performing pain assessment than physicians [23, 52]. On the other hand, questions about objective measures of pain (q5 and q13) were answered incorrectly by HCPs specially nurses. This difference might be due to their misinterpreting of the questions or their inability to differentiate between the two concepts.

A significant finding of this research is that knowledge and awareness of HCPs toward cancer pain and its management is satisfactory. This is consistent with numerous studies that showed adequate knowledge of cancer pain and pain management among HCPs [13, 35, 53]. This result probably reflects the better training programs and the emphasis put on pain management in oncology wards.

Noticeable deficiency in knowledge related to children's pain and pain management, particularly neonates was noted in the current study. HCPs' correct answer rate to questions related to this group of patients was the lowest. Likewise other studies described the ill preparation of HCPs to assess and manage children's pain [15, 16, 27, 50, 54, 55].

Consistent with other studies [46, 49], the percentage of correct answers was significantly higher in private with respect to public hospitals. This finding is not surprising and might be explained, in part, by the small size of private hospitals taking part in the survey. HCPs working at such hospitals, specifically nurses, are usually less stressed at work and can give more time to treatment of pain, not a priority at governmental hospitals. Moreover, good assessment and reporting of pain is part of quality criteria that private hospitals have to ascribe to. A study in Lebanon showed that accreditation improved quality of health care, particularly in documentation and translating theories into actions [56]. Aghaei Hashjin et al. reported an improvement in the performances of hospitals in Iran following implementation of a performance measuring program [57]. Moreover, auditing and monitoring coupled with education improved pain assessment and management in Canadian paediatric hospital [55]. In addition, public hospitals are larger, with higher patient traffic and a more stressful environment, which reflects the need to create and implement adequate clinical training courses in these hospitals.

Pharmacists and particularly PharmDs and clinical pharmacists are gaining more and more considerable role in clinical settings. Their knowledge and attitudes toward pain and pain management have not been explored extensively as other HCPs. In this study, one of the aims was to examine this neglected constituent of health care system. Despite the limited sample of pharmacists in this study, due to small number of pharmacist in clinical settings compared to nurses and physicians, the outcomes were interesting. Pharmacists' knowledge about pain and pain management was better than nurses but worse than physicians. Similar results reported by other studies showed that pharmacist' knowledge and attitudes toward pain and pain management were at comparable levels or slightly inferior to physicians [39, 41, 51]. Pharmacists can be instrumental and play a great role in the management of pain among patients at hospitals and clinics. Pharmacists have the potential to contribute significantly in any pain management program. A pain clinic lead by pharmacist-nurse team was effective at decreasing pain scores and lowering secondary referrals which ultimately decreased healthcare cost [58]. Pharmacists are underutilized in this regard, particularly community pharmacist who are in contact with many patients [51]. On the other hand, researches have shown that pharmacists can lead educational programs with positive results on the patients and the progress of their diseases [59].

In conclusion, patients are still hurting because of poor knowledge of healthcare providers toward pain and its management. Particularly nurses who are most in contact with the patient are in the best position to help and improve the patient healthcare. Pharmacists are also in a unique position to be instrumental in the effort of pain management, yet they are not utilized to their full potential. While physicians were better than nurses and scored the highest among HCPs surveyed in this study, they did have deficiencies in regard to knowledge of pain and pain management. Pain management is a complex, multidisciplinary effort and needs to be dealt with through several aspects. Education about pain management and offering of training programs are feasible and affordable ways to deal with these deficiencies and should be implemented by hospitals and clinics as a part of continuous medical education.

## Figures and Tables

**Table 1:** Demographic details of HCPs.

Characteristics	All HCPs *n* (%)
Gender	
Male	305 (46.1)
Female	357 (53.9)
Age (years)	
20–24	74 (11.4)
25–35	343 (52.7)
36–45	161 (24.7)
46–55	49 (7.5)
56–69	24 (3.7)
Discipline	
Physicians	198 (30.6)
Nurses	389 (60.1)
Pharmacists	60 (9.3)
Postgraduate degree (years)	72 (10.9)
Hospital	
Public	531 (80.2)
Private	131 (19.8)
Ward type	
Surgery	156 (26)
Medicine	116 (19.4)
Intensive care	64 (10.7)
Pediatric	87 (14.5)
Gynecologists & obstetrics	59 (9.8)
Others	117 (19.5)
Patients seen by week	101.09 ± 110.045 [0–1000]
Years qualified	
≤5 years	244 (39.7)
6–10 years	133 (21.6)
11–15 years	105 (17.1)
**>**15	133 (21.6)

**Table 2:** Overview of the questions.

Items in the questionnaire	Correct answers number (%)	Neither agree nor disagree answers number (%)
Q1 “Giving narcotics on a regular schedule is preferred over as needed (PRN) schedule for continuous pain”	456 (68.9)	50 (7.6)
Q2 “A patient should experience discomfort prior to giving the next dose of pain meds”	192 (29)	84 (12.7)
Q3 “When a patient requests increasing amounts of analgesics to control pain, this usually indicates that the patient is psychologically dependent”	167 (25.2)	123 (18.6)
Q4 “The most accurate judge of the intensity of the patient's pain is the patient”	473 (71.5)	95 (14.4)
Q5 “Staff can always pick up cues from children that indicate that they are in pain”	122 (18.4)	99 (15)
Q6 “Children cry all the time, therefore, diversional activities are indicated rather than actual pain meds”	150 (22.7)	134 (20.2)
Q7 “Because narcotics can cause respiratory depression, they should not be used in pediatric patients”	291 (44)	116 (17.5)
Q8 “The most suitable dose of morphine for a patient in pain is a dose that best controls the symptoms; there is no maximum dose”	310 (46.8)	74 (11.2)
Q9 “It may often be useful to give a placebo to a patient in pain to assess if he is genuinely in pain”	159 (24)	126 (19)
Q10 “For effective treatment of cancer pain it is necessary to continuously assess the pain and the efficacy of the therapy”	579 (87.5)	45 (6.8)
Q11 “It is a patient's right to expect total pain relief as a consequence of treatment”	522 (78.9)	73 (11)
Q12 “Lack of pain expression does not mean lack of pain”	471 (71.1)	74 (11.2)
Q13 “Estimation of pain by an M.D. or R.N. is as valid a measure of pain as a patient's self-report”	178 (26.9)	131 (19.8)
Q14 “Patients having severe chronic pain often need higher dosages of pain meds than patients with acute pain”	244 (36.9)	96 (14.5)

**Table 3:** Factors affecting respondents' knowledge towards pain.

Variable	OR (95% CI)	*p* value
Sex		
Female	Ref	
Male	0.811 (0.493–1.333)	0.408
Age (years)		
20–24	Ref	
25–35	0.901 (0.404–2.011)	0.799
36–45	0.786 (0.272–2.269)	0.656
46–55	0.495 (0.117–2.092)	0.339
56–69	0.466 (0.089–2.439)	0.366
Postgraduate degree		
No	Ref	
Yes	2.005 (1.081–3.720)	**0.027**
Discipline		
Physician	Ref	
Nurse	0.315 (0.184–0.537)	**<0.001**
Pharmacist	0.632 (0.162–2.464)	0.508
Patients seen by week	0.999 (0.996–1.001)	0.150
Years qualified		
≤5 years	Ref	
6–10 years	1.364 (0.771–2.412)	0.286
11–15 years	2.001 (0.962–4.159)	0.063
>15 years	2.036 (0.783–5.295)	0.145
Hospital		
Public	Ref	
Private	1.844 (1.099–3.094)	**0.021**
